# On the Integration of *In Silico* Drug Design Methods for Drug Repurposing

**DOI:** 10.3389/fphar.2017.00298

**Published:** 2017-05-23

**Authors:** Eric March-Vila, Luca Pinzi, Noé Sturm, Annachiara Tinivella, Ola Engkvist, Hongming Chen, Giulio Rastelli

**Affiliations:** ^1^Molecular Modelling & Drug Design Lab, Department of Life Sciences, University of Modena and Reggio EmiliaModena, Italy; ^2^Discovery Sciences, Innovative Medicines and Early Development Biotech Unit, AstraZeneca R&D GothenburgMölndal, Sweden

**Keywords:** drug repurposing, drug discovery, molecular modeling, chemogenomics, structure-based drug design, ligand-based drug design, machine learning, transcriptomics

## Abstract

Drug repurposing has become an important branch of drug discovery. Several computational approaches that help to uncover new repurposing opportunities and aid the discovery process have been put forward, or adapted from previous applications. A number of successful examples are now available. Overall, future developments will greatly benefit from integration of different methods, approaches and disciplines. Steps forward in this direction are expected to help to clarify, and therefore to rationally predict, new drug–target, target–disease, and ultimately drug–disease associations.

## Introduction

Drug repurposing (also known as drug repositioning) aims at identifying new uses for already existing drugs (Novac, 2013). In drug discovery, drug repurposing has gained an increasingly important role, because it helps to circumvent preclinical development and optimization issues, hence reducing time efforts, expenses and failures typically associated with the drug discovery process.

Over the years, biological and chemical information has been generated at an ever-increasing pace, marking the entrance in the so-called “big data” era (Costa, 2014). This offers the scientific community new opportunities to link drugs to diseases, although this relationship is indirect and relies on complex mechanisms of action. Therefore, a better understanding of the relationships between drugs and their targets, and between targets and diseases, is a key for drug repurposing. Unfortunately, we are still far from understanding the overall picture, partly due to the heterogeneity and incompleteness of the available data. However, computational methods offer valuable opportunities to create such links, as it will be illustrated below.

In this perspective, different computational methods and approaches are briefly presented, and their ability to complement and integrate each other in drug repurposing is discussed, which will certainly gain a foothold in the future.

## Computational Drug Repurposing Strategy Based on Transcriptional Signatures

Transcriptomic data can provide a list of over- and under-expressed genes in a biological system treated by a pharmacologically active compound. The perturbation of a biological system can be measured from genome wide transcriptional responses, and the drug induced transcriptional responses represent the signature of the compound activity on biological systems. These molecular transcriptional signatures can then be compared to establish therapeutic relationships between known drugs and new disease indications.

One of the most comprehensive and systematic approaches toward leveraging the transcriptional signature approach for drug repositioning is the Connectivity Map project (Lamb et al., 2006). The publicly funded CMap database^1^ initially contained profiles of 164 drugs and was later expanded to 1309 FDA-approved small molecules. These compounds are tested in five human cell lines, generating over 7000 gene expression profiles in the database. The cell perturbation profile of each drug in the reference collection contains, for each gene measured, a rank-based measure of the change in transcriptional activity after exposure to the drug compound, i.e., gene signatures. These signatures form the basis of comparing drugs mechanism of action at transcriptional level and have been successfully applied for drug repurposing in many examples. Chang et al. (2010) used CMap to identify new analgesic and antinociceptive properties of phenoxybenzamine, originally an anti-hypertensive drug. Subsequent testing using a rat inflammatory model validated the analgesic activity. In contrast with CMap gene signatures, biclustering methods were applied to CMap to group coregulated genes with the drugs they respond to Iskar et al. (2013). This led to the identification of vinburnine, a vasodilator, and sulconazole, a topical antifungal, as interesting cell cycle blockers for cancer therapy.

## Network-Based Drug Repurposing

In recent years, network-based computational biology has attracted increasing attention. It aims at organizing the relationships among biological molecules in the form of networks to find newly emerged properties at a network level, and to investigate how cellular systems induce different biological phenotypes under different conditions. In the network pharmacology framework, a network can be depicted as a connected graph, where each node can represent either an individual molecular entity (e.g., a drug), its biological target, a modifier molecule within a biological process, or a target pathway, while an edge represents either a direct or indirect interaction between two connected nodes. Ultimately, both the efficacy and the toxicity of a drug are a consequence of the complex interplay among different cellular components. A system-scale perspective is therefore needed to aid modern drug discovery, especially for complex diseases, which are known to be caused by perturbation of biological networks.

Network-based analysis has become a widely used strategy for computational drug repositioning. Hu and Agarwal (2009) created a disease-similarity network using publicly available gene expression profiles from NCBI Gene Expression Omnibus (GEO)^2^ and integrated this network with molecular profiles and knowledge of drugs and drug targets to infer drug repositioning opportunities and suggest molecular targets and mechanisms underlying drug effects. Jin et al. (2012) developed a novel method to repurpose drugs for cancer therapeutics by leveraging off-target effects that may affect important cancer cell signaling pathways. The off-target effects of drugs on signaling proteins were identified by using a hybrid model composed of a network component called cancer-signaling bridges and Bayesian factor regression model.

## Ligand-Based Approaches in Drug Repurposing

Ligand-based approaches are based on the concept that similar compounds tend to have similar biological properties. In drug repurposing, these methods have been extensively used to analyze and predict the activity of ligands for new targets. Public databases of bioactive molecules, such as PubChem, ChEMBL, and DrugBank contain information retrieved and manually curated from literature data (Wishart et al., 2006; Gaulton et al., 2017; Wang et al., 2017). These databases represent a huge and ever-growing reservoir of chemical and biological information such as binding affinity, cellular activity, functional and ADMET data. Recent advances in drug repurposing include the release of databases focused on repurposed drugs, failed drugs, their therapeutic indications, and bioactivity data (Brown and Patel, 2017; Shameer et al., 2017).

One advantage of applying these approaches to drug repurposing is that the number of publicly accessible compound records (more than hundred millions provided only by PubChem) is far greater than the number of deposited protein crystal structures (as of today, less than 150,000 in the Protein Data Bank) (Berman et al., 2000; Wang et al., 2017). On the other hand, ligand-based methods obviously depend on the chemical space coverage of already known molecules. Moreover, a high overall similarity does not necessarily guarantee activity on a secondary target, since local structural divergences in chemical scaffolds can lead to “activity cliffs” (Stumpfe and Bajorath, 2012). This limitation, however, will eventually be overcome by the increase of structural diversity in bioactivity databases (Hu and Bajorath, 2013).

Recently, a 2D ligand-based similarity analysis of ChEMBL combined with support vector machine models and analysis of 3D structural information of protein–ligand complexes, identified a promising set of target combinations and associated ligands within the Hsp90 interactome, which are particularly suitable for multitarget drug design (Anighoro et al., 2015). Another ligand-based method correctly predicted 23 new drug–target associations using the similarity ensemble approach (Keiser et al., 2009). Pharmacophore screening has also been a valuable strategy for drug repurposing (Liu et al., 2010). In this approach, a drug can be represented as a set of pharmacophoric features which can subsequently be used to interrogate chemical compound databases to provide compounds with different scaffolds.

Complementing different levels of ligand description increases the chances of identifying new repurposing possibilities. For example, Vasudevan et al. (2012) used 3D shape-based descriptors to compare approved drugs with a set of H1 receptor antagonists. Thirteen of the 23 tested drugs selectively inhibited histamine-induced calcium release by acting at the H1 receptor level. Interestingly, these drugs would not have been detected with 2D similarity searching (Vasudevan et al., 2012). Furthermore, Mervin et al. (2015) demonstrated how the inclusion of inactive data improved early recognition abilities in statistical prediction models. On different grounds, predictive models built upon disease feature descriptors, large-scale drug–target and target–disease associations showed performance improvements in predicting new drug–disease links (Iwata et al., 2015; Sawada et al., 2015). In particular, it was shown that chemical similarity and phenotypic similarity are complementary to each other, and that integrating predictions from both methods is beneficial (Sawada et al., 2015).

## Ligand-Based Chemogenomics and Machine Learning in Drug Repurposing

A variety of *in silico* approaches have been applied in ligand-based chemogenomic campaigns (Mestres et al., 2006; Bender et al., 2007; Gregori-Puigjané and Mestres, 2008). During the last years, machine learning algorithms, which span from the older but still attractive Bayesian classifiers to the more advanced support vector machines, have become increasingly popular to assist the drug repositioning process (Bender et al., 2007). Methods such as deep learning and multi-task learning have been successfully used in chemogenomic benchmark studies (Unterthiner et al., 2014). Moreover, matrix factorization methods offer the opportunity to combine bioactivity data with other information, such as disease information, in one framework (Zhang et al., 2014). On a different line, other techniques inspired by e-commerce websites have shown interesting results in identifying new drug–target associations (Alaimo et al., 2016). In the study, the technique relies on a network-based inference algorithm and a drug–target bipartite graph extracted from DrugBank. It was shown that the algorithm performed better in predicting new drug–target associations when target and drug similarities are considered.

Given the versatility in their use and their computational efficiency, machine-learning approaches will likely continue to play a prominent role in *in silico* chemogenomics. Despite many papers have described test cases and various types of method development, there is still a lack of published success stories that employed ligand-based chemogenomics modeling in drug repurposing.

## Structure-Based Approaches in Drug Repurposing

It is established that the similarity principle observed for ligands applies also to proteins. Proteins with similar structures are likely to have similar functions and to recognize similar ligands. In the field of drug repurposing, protein comparison is used as a method to identify secondary targets of an approved drug (Ehrt et al., 2016).

From a global point of view, proteins can be compared by sequence similarity. Protein sequences have been used to build phylogenetic trees, the most popular of which is represented by the kinome (Manning et al., 2002). In this tree, proteins of the same family are prone to have related functions and also to recognize related substrates or ligands, such as for example dual inhibitors of epidermal growth factor receptor (EGFR) and epidermal growth factor receptor B2 (ErbB2) (Zhang et al., 2004). Modern methods to perform multiple-sequence alignments, such as BLAST, are nowadays widely used and available through web-servers. It is important to note that small differences localized at key positions, such as those occurring in correspondence of the gatekeeper residue of protein kinases or of other oncogenic mutations, may have a huge impact on ligand binding (Huang and Fu, 2015). Hence, local differences in globally conserved protein sequences should be given careful consideration. Moreover, a study based on the similarity ensemble approach showed that similar ligands were able to bind proteins with distantly related sequences (Keiser et al., 2007). Overall, local binding site similarities can be more important than global similarities to determine polypharmacology and drug repurposing (Jalencas and Mestres, 2013b; Anighoro et al., 2015).

In identifying unknown targets of known ligands, sequence alignments perform well when proteins share a high degree of sequence identity, whereas local protein comparison performs better when proteins share low sequence identity (Chen et al., 2016). Detecting local similarities by comparing protein binding sites has become increasingly important (Ehrt et al., 2016). Binding site identification and comparison are commonly performed by scanning the protein surface in order to identify cavities (Laurie and Jackson, 2006) and then by calculating descriptors of different nature useful to derive a similarity score.

It is important to note that several approaches and algorithms for binding site comparison have been put forward, but none of them appears to be devoid of failures or limitations (Ehrt et al., 2016). Notwithstanding, binding site similarity has proven a valuable tool in a number of studies. For example, a study carried out by Defranchi et al. (2010) used a binding site comparison method to predict the cross-reactivity of four protein kinase inhibitors with Synapsin I. These discoveries were supported by sub-micromolar affinities of the kinase inhibitors for Synapsin I. Interestingly, binding site similarity and other molecular modeling techniques were used in combination to uncover new targets of the drugs entacapone and tolcapone (Kinnings et al., 2009). The study started from a large set of similar binding sites, which was further finalized by simulating the binding mode of entacapone and tolcapone using docking. Proteins for which ligands gave the best docking scores were prioritized and further experimentally validated.

It is worth mentioning that ligand binding modes, when available, are a strong asset in the process of identifying new targets. One way to model the molecular recognition is to focus on target–ligand interactions. This can be achieved with various methods, such as structure-based pharmacophores or interaction fingerprints. When the structure of a protein–ligand complex is not available, one can use computational methods to predict hot spots in the binding site (Hall et al., 2015). Another approach joining ligand information to protein environments uses the concept of chemoisosterism (Jalencas and Mestres, 2013a). Chemoisosterism can be defined as the property of two protein environments to bind the same molecular fragment, and can shed light into the inherent cross-pharmacology between protein targets. The degree of chemoisosterism was found to be related to the polypharmacology of chemical fragments (Jalencas and Mestres, 2013b). This approach allows the creation of interaction networks connecting chemical fragments to chemoisosteric protein environments. These networks, complemented with target–disease associations, constitute attractive starting points for drug repurposing efforts.

Based on similar concepts, a method for interrogating large data sets of proteins (as large as the PDB) with highly customizable geometric patterns as searching templates was recently described (Inhester et al., 2017). This method was able to identify chemoisosteric protein environments binding the uracil moiety of uridine diphosphate from a query built with deoxythymidine.

Structure-based methods are obviously dependent on the availability of crystallographic structures of protein–ligand complexes. Resolution and sensitivity to atomic coordinates impact the level of details that one can use to model a binding site. While crystallographic structures represent a static model of a protein, other pockets may appear upon conformational changes. Detecting those cryptic sites has become an emerging field of research, because it may provide additional options in drug repurposing. In fact, cryptic allosteric sites may be useful to gain selectivity, explore new chemical spaces for drug design, and establish drug–target associations beyond the more commonly explored orthosteric site. For instance, Markov models have been applied in combination with experimental assays using a chemical probe to uncover cryptic allosteric sites of TEM-1 β-lactamase (Bowman et al., 2015). Overall, uncovering new allosteric sites in proteins may provide far more opportunities to repurpose drugs than is currently recognized.

## Molecular Docking

Molecular docking is a versatile tool used to predict the geometry and to score the interaction of a protein in complex with a small-molecule ligand (Kitchen et al., 2004). Therefore, these methods can be used to predict if a given drug is potentially able to bind other targets. Docking studies have been successfully exploited in drug repurposing, as reported in many recent studies (Kinnings et al., 2009; Li et al., 2011; Dakshanamurthy et al., 2012). In this context, virtual screenings can be performed either by docking a known drug into a large set of different target structures, or by docking a database of approved drugs into one intended specific target. Molecular docking is in fact a convenient and fast method to screen large libraries of both ligands and targets, with a full range of sampling options (Kitchen et al., 2004), and is obviously restricted to studies in which a 3D structure of the target is available through crystallography, nuclear magnetic resonance (NMR), or comparative models. It should be noted that docking methods still have drawbacks and limitations, mainly arising from the use of approximate scoring functions and imperfect binding mode placement algorithms. Often these problems can be overcome by post-processing docking results with more accurate scoring functions and/or other criteria (Sgobba et al., 2012).

In the study of Li et al. (2011), docking methods have been successfully exploited as a stand-alone method in drug repurposing, by docking the drugs of the DrugBank database into 35 crystal structures of MAPK14. The study identified the chronic myeloid leukemia drug nilotinib as a potential anti-inflammatory drug with an *in vitro* IC_50_ of 40 nM (Li et al., 2011).

Docking is notably well suited for either drug-based and target-based drug repurposing, as reported in the results of the work of Dakshanamurthy et al. (2012), where an anti-parasitic drug was successfully tested as an anti-angiogenic vascular endothelial growth factor receptor 2 (VEGFR2) inhibitor, and a new connection was discovered between previously untargeted Cadherin-11, implied in rheumatoid arthritis, and cyclooxygenase-2 (COX-2) inhibitor celecoxib.

It is important to note that docking, despite its limitations, is a well-established and experimentally validated approach for predicting new drug–target associations. Once integrated with ligand-based methods and other available information about target–disease associations, it constitutes a powerful approach to repurpose (newly) targeted drugs for a specific disease.

## Integrating Different Approaches and Future Directions

**FIGURE 1:**
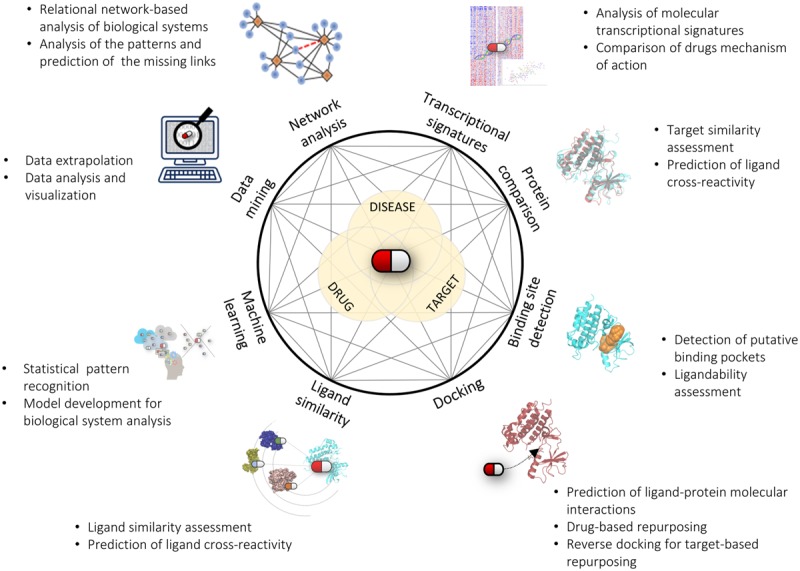
**Connecting drugs, targets and diseases with *in silico* methods.** Like in a network representation, the integration of different computational methods and approaches will greatly help us advance our understanding and prediction of the complex interplay between drugs, targets, and diseases.

The goal of drug repurposing is to uncover new links between drugs and diseases, most commonly *via* targets. As illustrated in the previous sections, computational predictions followed by experimental assessment have been successfully used to identify new drug repurposing possibilities. As always, each computational method has its own field of applicability, drawbacks and limitations. One should be aware of the fact that none of these methods alone will be sufficiently able to disclose (or even model) the complex interplay between drugs, targets and diseases. Therefore, we are left with the possibility of using one or more computational approaches to “navigate” through the wealth of available information and hopefully find “clues” solid enough to justify a repurposing hypothesis worth of experimental investigation. The choice of the most appropriate method(s) will basically depend on the nature of the problem to solve and on the type, quality, and quantity of information available on that problem in the literature or in public or proprietary databases. Unfortunately, information is often fragmented, and generally reflects only a single or few aspects of a much more complicated story. Future efforts should be more thoroughly directed toward disclosing hubs and links of the complex network that relates drugs, targets and diseases. Integrating the huge and heterogeneous amount of available data (chemical, biological, structural, clinical) into a unified workflow is obviously a challenging task. In this respect, the integration and use of different computational methods as shown above will provide valuable opportunities to extend the domain of applicability of each method and more thoroughly exploit information coming from different sources (**Figure 1**). Likewise, this will greatly benefit from better integration of multidisciplinary work. A network-based approach built upon these considerations will likely provide new routes to navigate through all the potential links between drugs and diseases, thus creating new opportunities for drug repurposing and drug discovery in general.

## Author Contributions

All authors contributed in writing and editing the manuscript. EM-V, LP, NS and AT contributed equally. GR conceived the study and coordinated the writing.

## Conflict of Interest Statement

The authors declare that the research was conducted in the absence of any commercial or financial relationships that could be construed as a potential conflict of interest.

## Footnotes

1 http://www.broadinstitute.org/cmap

2 http://www.ncbi.nlm.nih.gov/geo/
